# The Campanian Ignimbrite Eruption: New Data on Volcanic Ash Dispersal and Its Potential Impact on Human Evolution

**DOI:** 10.1371/journal.pone.0065839

**Published:** 2013-06-17

**Authors:** Kathryn E. Fitzsimmons, Ulrich Hambach, Daniel Veres, Radu Iovita

**Affiliations:** 1 Department of Human Evolution, Max Planck Institute for Evolutionary Anthropology, Leipzig, Germany; 2 Chair of Geomorphology, Laboratory for Palaeo- and Enviro-Magnetism, University of Bayreuth, Bayreuth, Germany; 3 Institute of Speleology, Romanian Academy, Cluj-Napoca, Romania; 4 Faculty of Environmental Sciences, Babes-Bolyai University, Cluj-Napoca, Romania; 5 MONREPOS Archaeological Research Centre and Museum for Human Behavioural Evolution, RGZM, Neuwied, Germany; New York State Museum, United States of America

## Abstract

The Campanian Ignimbrite (CI) volcanic eruption was the most explosive in Europe in the last 200,000 years. The event coincided with the onset of an extremely cold climatic phase known as Heinrich Event 4 (HE4) approximately 40,000 years ago. Their combined effect may have exacerbated the severity of the climate through positive feedbacks across Europe and possibly globally. The CI event is of particular interest not only to investigate the role of volcanism on climate forcing and palaeoenvironments, but also because its timing coincides with the arrival into Europe of anatomically modern humans, the demise of Neanderthals, and an associated major shift in lithic technology. At this stage, however, the degree of interaction between these factors is poorly known, based on fragmentary and widely dispersed data points. In this study we provide important new data from Eastern Europe which indicate that the magnitude of the CI eruption and impact of associated distal ash (tephra) deposits may have been substantially greater than existing models suggest. The scale of the eruption is modelled by tephra distribution and thickness, supported by local data points. CI ashfall extends as far as the Russian Plain, Eastern Mediterranean and northern Africa. However, modelling input is limited by very few data points in Eastern Europe. Here we investigate an unexpectedly thick CI tephra deposit in the southeast Romanian loess steppe, positively identified using geochemical and geochronological analyses. We establish the tephra as a widespread primary deposit, which blanketed the topography both thickly and rapidly, with potentially catastrophic impacts on local ecosystems. Our discovery not only highlights the need to reassess models for the magnitude of the eruption and its role in climatic transition, but also suggests that it may have substantially influenced hominin population and subsistence dynamics in a region strategic for human migration into Europe.

## Introduction

The Phlegrean Fields super-eruption and caldera collapse that produced the Campanian Ignimbrite (CI)/Y-5 tephra, which took place 39.28±0.11 ka [1], was one of the most explosive eruptions affecting Europe in the late Pleistocene [2], in terms of both eruption magnitude and volume of volcanic ejecta. The distal ashfall of CI tephra was widely distributed from its source vent near Naples in southern Italy [3], [4], eastward over the Balkans [5] and Black Sea [6] to the Russian plain more than 2200 km distant [7]–[9], and over 1000 km southward to the north African coast [10]–[12]. The CI tephra thereby provides a powerful chronostratigraphic marker horizon for palaeoclimatic and archaeological records during Marine Isotope Stage (MIS) 3. Moreover, the timing of this eruption coincides with the onset of the cold, dry climatic phase Heinrich Event 4 (HE4) [13], [14]. It has been proposed that the conjunction of these two events may have triggered a positive feedback cycle affecting global, and particularly European, climates for hundreds or even thousands of years [11], [15]. Significantly, the timing of the eruption, and of the extreme environmental conditions during HE4, also coincides with significant changes in the archaeological record in Europe; specifically, the arrival of anatomically modern humans (AMHs) [16]–[18], a substantial shift in hominin lithic technology [18]–[20], and the disappearance of Neanderthals from the continent [21], [22]. However, the role of the CI super-eruption in the interaction between sudden climatic change, the demise of the Neanderthals, and their replacement by AMHs, remains a matter of hypothesis [10], [15], [23].

The CI super-eruption took place in the Phlegrean fields of southern Italy [2], and has been dated based on a composite ^40^Ar/^39^Ar age of 39.28±0.11 ka obtained from proximal ignimbritic deposits [1]. The volcanic cataclysm involved a two-step eruption, consisting of an initial phase with a volcanic column at least 40 km high, followed by collapse and creation of the caldera, a rejuvenated volcanic column, and widespread ignimbritic deposition extending at least 1500 km^2^ from the eruption point [9], [24] with sufficient force to ascend the surrounding topography to over 1000 m altitude [25]. A peak in sulphate concentration within the GISP2 Greenland ice core, only slightly lower than the Icelandic Z2 ash or Toba eruptions, was correlated with the CI eruption [26]. However, subsequent investigations failed to identify tephra shards at the same depth within the core or a comparable peak within other Greenland ice core records, urging caution when such correlations are proposed based on only geochemical proxies [27], [28].

**Figure 1:**
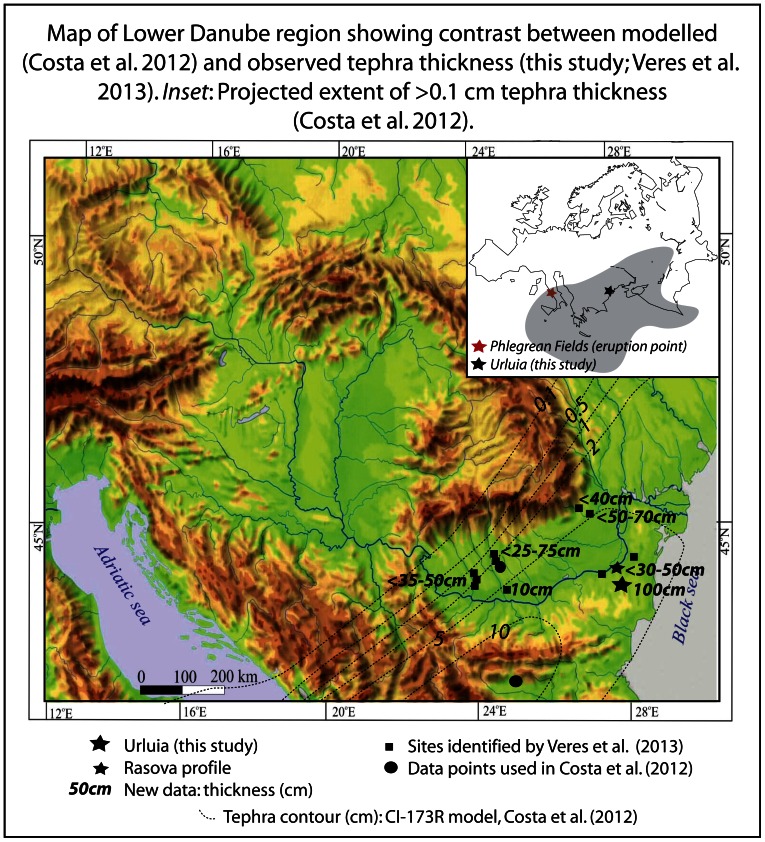
Map of the Lower Danube region showing the contrast between modelled [12] and observed thickness of the CI tephra (this study; [5]). Inset shows the projected extent of >0.1 cm tephra thickness, based on data from [12].

Interaction of the volcanic column with high altitude wind currents transported finer-grained volcanic particles (<250 µm) northeastward and southward, as far as the Russian Plain, eastern Mediterranean, Black Sea and north Africa (Figure 1a) [3], [6], [12]. Recent modeling of tephra transport [12], based on measured tephra thickness from several sites in the Balkans, Russian Plain and from eastern Mediterranean sea cores, suggested that the magnitude of the eruption was more than twice that of previous estimates. The modeling study also predicted the thickness of ash cover for regions beneath the ash plume for which data previously did not exist. Depending on the model parameters, the thickness of tephra deposited across the Balkans proximal to the Adriatic Sea should average 5–10 cm, decreasing to 2–5 cm in eastern Europe (Romania, Moldova, southern Ukraine), with the plume tapering northeastwards over the Russian Plain (Figure 1a) [12]. However, the model output, while providing a useful estimate of the magnitude of the eruption, contains no data points spanning the 1500 km between the Balkan sites and the Don River on the Russian Plain (Figure 1b).

Significant thick deposits corresponding to the CI tephra have recently been identified in southern Romania [5], [29]–[31], and could provide important information for better constraining the magnitude of the eruption and its likely environmental impact. Moreover, elucidating the nature and spatial extent of the tephra fallout, as well as its likely forcing on regional climates, is also important for assessing the regional impact on hominin populations and their resilience during this critical period of time. In this paper we provide significant new data from the loess steppe environments of Dobrogea in southeastern Romania, a narrow land corridor between the Danube River and Black Sea. Our data not only expand on the critical mass of new data points from a region previously unaccounted for in the models of tephra dispersal, but also indicates an average tephra thickness substantially greater than the models predict (Figure 1b). These observations could present major implications for the magnitude of the eruption and its role within climate and environmental feedbacks.

The timing of the CI super-eruption coincided with the onset of Heinrich Event 4 (HE4) [32], [33], a short-lived stadial (Greenland Stadial 9, GS9) associated with enhanced ice-rafting in the North Atlantic Ocean and cold, dry conditions over Europe [34], [35]. This was followed by a series of centennial- to millennial-scale warm interstadials (separated by stadials), of which Greenland Interstadial 8 (GIS8) [14] was the longest and most pronounced event, occurring at the transition from Middle to Upper Pleniglacial [36]. Climatic conditions across the northern hemisphere during HE4 were generally cool and dry [35] due to a southward shift of the polar front driven by the collapse of the thermohaline circulation in the northern oceans. Palynological records from the Lago Grande di Monticchio in southern Italy, not far from the Phlegrean Fields, suggest abrupt cooling and more arid conditions during HE4 immediately following CI tephra deposition [32], [37], [38]. Indications of cooler climates during HE4 within existing eastern European records are not as clearly defined as in the Italian and other Mediterranean palaeoclimate archives [39]–[41]. In this respect, the sedimentary deposits of the temperate eastern European loess steppe are particularly poorly understood [41], [42]. Aside from the limitations of existing records to preserve evidence of HE4 intensity and its connection to the CI-super-eruption, the potential feedback link is difficult to establish beyond mere hypothesis given existing datasets, often of low analytical and consequently low temporal resolution [11]. However, comparable attempts to identify a causal link between volcanic winters and climatic change in the case of the earlier Toba super-eruption in Sumatra have so far proven similarly inconclusive [43]–[46], although recent chronological constraints have linked the Toba eruption with a substantial cooling event [46]. A better understanding both of the nature and intensity of climate change during HE4 across Europe, the temporal connection with the CI, and potential for establishment of positive climate feedbacks, is clearly necessary to establish the significance of the CI super-eruption within the global climate system.

The timing and impact of the CI super-eruption is of particular interest and relevance to human evolution in Europe, since it coincides with a substantial shift in the archaeological record, associated with the arrival of AMHs to the continent [16]–[18], and the demise of the Neanderthals [21], [22]. Archaeological records within the CI ashfall zone presently provide an inconsistent picture of the eruption’s influence on hominin occupation [10], [47], [48]. In southern Italy, the region closest to the volcanic cataclysm, both open air and rock shelter sites preserve a significant hiatus in occupation during the period immediately following the CI eruption [3], [49], [50]. This indicates widespread abandonment of habitation sites in the region for some hundreds, if not thousands, of years following the eruption [11], [48]. The archaeological record from further afield is contradictory. Several rock shelters in the Balkans and northern Africa preserve CI tephra shards within their stratigraphy, yet interpretations of archaeological records within these sites postulate no significant disruption of the archaeological record and conclude that the CI super-eruption did not affect hominin populations there [10]. Conversely, the open air site of Kostenki 14 on the Russian Plain preserves an archaeological assemblage suggesting sudden catastrophic destruction of a human settlement [51], despite the fact that the tephra layers in that sequence appear to be redeposited [52]. Moreover, thick deposits of tephra within the Montenegran cave site of Crvena Stijena clearly divide the Middle and Upper Palaeolithic [53].

The localised impacts of large volcanic eruptions are not exclusively climatic. Chemical reactions between acidic volatile volcanic gases, and atmospheric and soil moisture, produce acid rain and soil acidification in ashfall zones, contamination of freshwater systems, and fluorosis of herbivores ingesting contaminated vegetation, with associated effects on humans dependent on affected ecosystems [54], [55]. Therefore, in considering the impact of the CI super-eruption on human evolution within an ashfall zone, more than just the influence of climate comes into play. In this sense, accurate data on tephra distribution and thickness within the ashfall region becomes critically important.

The lower Danube River valley and its major tributaries in eastern Europe has long been proposed to represent one of the major migration routes for AMHs into Europe [17], [56]. Yet this is precisely the region most likely to have been affected by the ecological impacts of the CI ashfall and related impacts on the regional ecosystem. Consequently, at this stage not only is it unclear what impact the CI eruption might have had on hominin occupation; the conjunction between hypothesized migration routes into Europe, potential interaction between hominin species, and tephra deposition may well have intensified this impact.

In this paper we present new data from a tephra deposit in the CI distal ashfall region of the lower Danube basin in southeast Romania, consolidated with recently published data confirming widespread CI tephra from other sites nearby [5]. Based on the characteristics of the deposit, we show that the CI tephra was deposited not only rapidly but also more thickly than predicted, 1200 km east of the eruption. We hereby propose that models of the magnitude of the eruption be reassessed using the new data points from this distal region for which data previously did not exist. Although we do not remodel the volcanic event within this paper, we do hypothesise that the eruption was substantially more explosive than previously estimated. We speculate on the potential impact of such rapid, thick deposition of ash on hominin populations in this region, and for the spatial variability of this impact, both climatically and directly on the ecosystem. Our hypotheses not only hold significant implications for human evolution within Europe in general, but also highlight the complexity and potential vulnerability of hominin dispersals and occupations at varying scales across the continent at a critical time in human evolution.

## Results

### The Tephra Deposit at Urluia, Southeast Romania

The Quaternary-uplifted Dobrogea plateau, in southeastern Romania, preserves some of the thickest loess deposits in the lower Danube basin [41], [57], [58]. The loess is derived mostly from aeolian transport of fluvial silts associated with glaciers at the head of the Danube catchment [59]–[61], with minor components from the Saharan Desert to the south [62] and Russian Plain and Caspian Basin to the east [60], [63]. Loess in Dobrogea typically occurs as plateau deposits, characterised by aeolian draping over low angle slopes. Multiple phases of intensified deposition and pedogenesis, typically associated with glacial and interglacial phases respectively, are preserved in the form of loess-paleosol sequences [41], [64]. Sedimentary differentiation between paleosols and overlying loess, and in the case of this study, loess and tephra, enables low angle palaeotopography and loess palaeokarst to be clearly distinguished.

**Figure 2:**
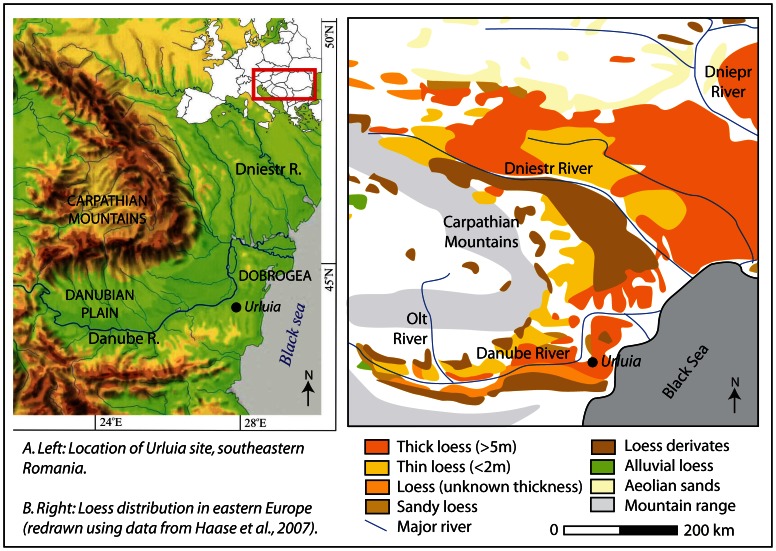
Location of Urluia loess deposit, southeastern Romania (a), and distribution and thickness of loess deposits in the lower Danube region (b; redrawn using data from from [57]).

In this paper we investigate an especially thick deposit of CI tephra from the Urluia Quarry site, a substantial exposure of loess and limestone basement rocks located on the Dobrogea loess plateau (Figure 2a). Urluia Quarry lies approximately 15 km south of the Danube River, within a zone of particularly thick loess (Figure 2b). At this site, a thick (20+ m) sequence of loess-palaeosol packages overlies the uplifted Cretaceous-Tertiary-age limestone of the Dobrogea plateau [65]. The present land surface is predominantly horizontal, dipping more steeply on the western margins of the quarry into a small tributary of the Danube. Palaeotopography visible from the differentiation of stratigraphic units indicates the land surface to have changed slightly with time, most likely due both to tectonic activity and surface geomorphic processes. Karst phenomena such as dolines, infilled cave conduits and karst springs are also documented on a limited scale.

**Figure 3:**
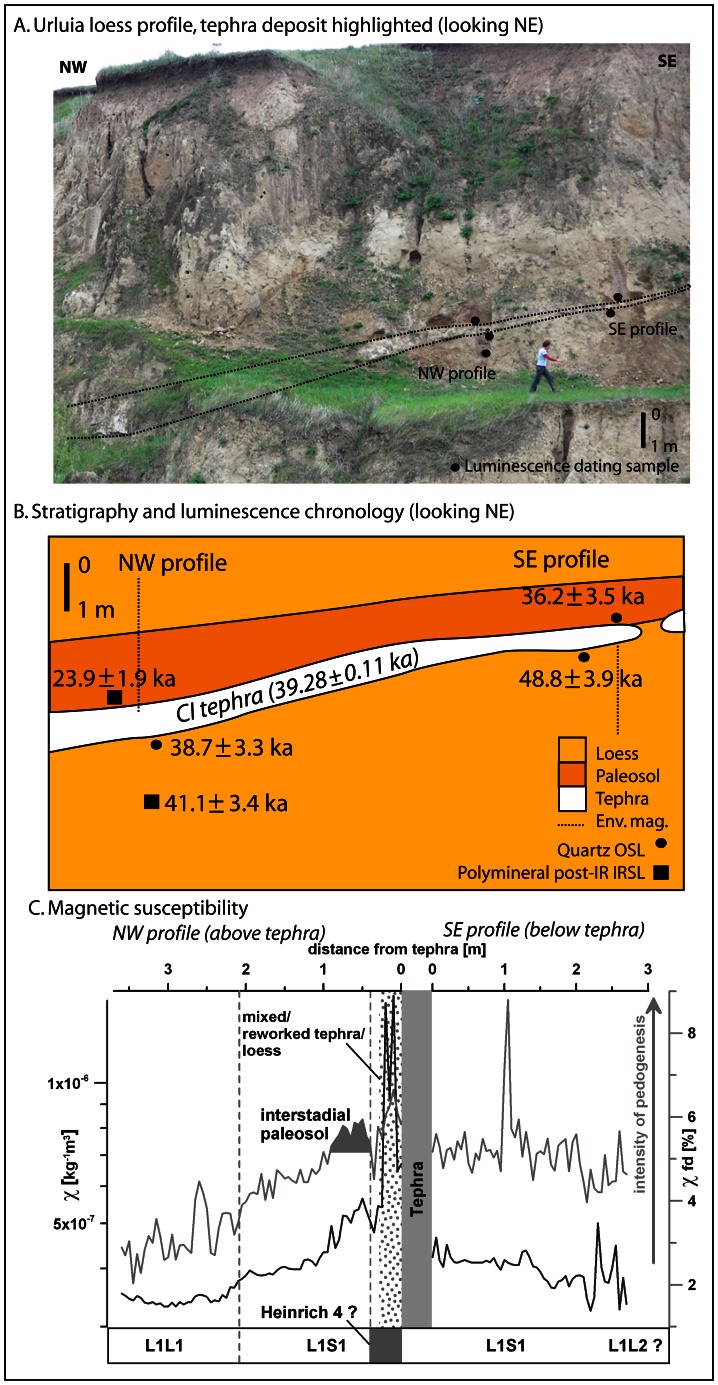
The tephra deposit at Urluia. a. Photograph of the site. b. Stratigraphic section, showing luminescence ages and methods used. c. Magnetic susceptibility of the northwest and southeast loess profiles, showing frequency dependent (χ_fd_) and magnetic low field susceptibility (χ_,_) as functions of stratigraphy below and above the tephra layer. Bulk χ reflects the concentration of the magnetisable fraction in sediments; χ_fd_ reflects the relative amount of pedogenetic and diagenetic neo-formation of ultrafine Fe-particles.

The tephra at Urluia occurs as a distinct, pale whitish, coarser-grained, fine sand-sized unit within the buff-coloured silty loess of the uppermost loess-paleosol package that represents the last full glacial cycle (Figure 3a). The palaeotopography of the tephra exposure suggests a gentle depression in the central part of the land surface, which was subsequently infilled by tephra and then by loess. Given that the upper limit of the last interglacial paleosol appears largely horizontal, the palaeodepression filled in by the tephra may have developed after MIS 5. The thickness of the tephra ranges between ca. 0–100 cm, and is thinner on the upper, steeper parts of the palaeotopographic slope, and thickest towards the base of the depression (Figure 3a). The ash was analysed using geochemical and geochronological methods in order to determine its provenance and assess its depositional age. Sedimentological and environmental magnetic studies were used to establish the mode of deposition and the relative stratigraphic context.

### Analysis and Provenance of the Tephra at Urluia

**Figure 4:**
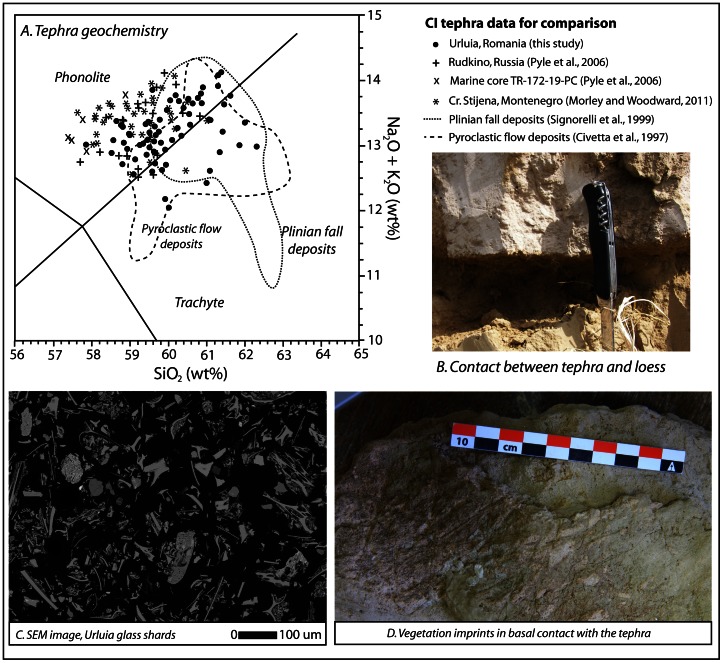
Characteristics of the tephra at Urluia. a. Geochemical profile of the Urluia tephra compared with CI data from Italy, eastern Europe and Mediterranean Sea cores [4], [8], [53], [66]. b. Photograph of the contact between the tephra and underlying loess deposits. c. Photomicrograph from scanning electron microscope (SEM) of tephra shards. d. Vegetation imprints at the basal contact of the tephra with the loess (looking upward), collected from the exposure at Urluia.

The tephra was investigated in detail at two separate locations along the exposed section (“northwest” and “southeast”; Figure 3b). The provenance of the tephra was fingerprinted based on the chemical analyses of two ash samples collected from the base and top of the unit at the northwest profile where the ash is thicker. Analyses of major oxide concentrations from isolated glass shards yield consistent phonolite/trachyte compositions (Table 1, Table S1 in File S1; Figure 4a) consistent with the CI glass shard chemical composition [4], [8], [66]. The major oxide concentrations measured from both the top and bottom of the tephra (up to 40 grains measured for each sample) are indistinguishable from one another, indicating correspondence with the same eruptive event. The SiO_2_, K_2_O, Na_2_O, CaO and FeO compositions average 58.76–59.95 wt %, 7.10–7.53 wt %, 5.24–6.08 wt %, 1.77–1.83 wt % and 2.75–2.86 wt % respectively (Table 1). The geochemical composition correlates not only with proximal Plinian fall and pyroclastic flow deposits associated with the CI super-eruption in Italy [4], [66] but also with distal CI tephra deposits from the Eastern Mediterranean and Russian Plain [8] the Crvena Stijena archaeological site in Montenegro [53], and from other CI tephra occurrences in Romania [5].

**Table 1:** Geochemical analyses of major and minor element concentrations for tephra samples URL1 and URL2.

Oxide	URL1 (base of tephra)	URL2 (top of tephra)
	Average ± σ (wt %)	Average ± σ (wt %)
Na_2_O	6.08±0.87	5.24±1.56
SiO_2_	59.95±0.99	58.76±3.87
K_2_O	7.10±0.61	7.53±2.04
CaO	1.77±0.24	1.83±0.47
FeO	2.86±0.13	2.75±0.73
MgO	0.38±0.10	0.42±0.19
Al_2_O_3_	18.48±0.21	18.61±2.65
P_2_O_5_	0.05±0.04	0.09±0.06
TiO_2_	0.41±0.04	0.36±0.10
MnO	0.21±0.04	0.17±0.07
Cl^-^	0.69±0.13	0.54±0.23

The age of the tephra deposit at Urluia was constrained using luminescence dating performed on tephra-bracketing samples collected from the surrounding loess, from the northwest and southeast profiles along the exposed section (Figure 3b). Three samples were collected from below the tephra, and two collected from the overlying loess. The northwest profile yielded two ages below the tephra of 38.7±3.3 ka and 41.1±3.4 ka, in correct stratigraphic order, and an age from a paleosol horizon overlying the tephra of 23.9±1.9 ka (Table 2). The age of the uppermost sample suggests an unconformable boundary with the underlying tephra, although additional chronological analyses are required to clarify these aspects. The southeast profile yielded an age for the loess underlying the tephra of 48.8±3.9 ka, and for the overlying loess of 36.2±3.5 ka (Table 2). The significantly older age of the underlying loess compared with the tephra at this point may be accounted for by erosion of the loess prior to tephra deposition, although equally this age lies within 2σ of the other underlying sample and may be contemporaneous. All underlying ages, accounting for uncertainties, are older than the CI tephra. The two overlying ages are younger. These results corroborate the previously known age of proximal tephra in Italy of 39.28±0.11 ka [1], as well as those obtained for other deposits interbedded with the CI in Romania [30].

**Table 2:** Luminescence dating data and age estimates for the CI tephra at the Urluia site.

Sample code	Depth (m)	D_e_ (Gy)	σ (%)	Attenuated dose rates (Gy/ka)			Total dose rate (Gy/ka)	Age (ka)
				β	γ	Cosmic		
*L-EVA1091*	9.5±0.1	*114±2*	7	2.01±0.20	1.67±0.17	0.07±0.01	4.77±0.37	*23.9±1.9*
L-EVA1089	9.5±0.1	149±7	17	1.89±0.19	1.67±0.17	0.07±0.01	4.12±0.35	36.2±3.5
*CI TEPHRA*								
L-EVA1028	9.6±0.1	167±6	14	2.22±0.22	1.58±0.16	0.07±0.01	4.31±0.33	38.7±3.3
*L-EVA1029*	10.2±0.1	*193±4*	7	2.05±0.21	1.54±0.15	0.07±0.01	4.70±0.38	*41.1±3.4*
L-EVA1090	9.5±0.1	199±5	6	1.93±0.19	1.67±0.17	0.07±0.01	4.08±0.31	48.8±3.9

Samples analysed by OSL are shown in plain text; those analysed using the post-IR IRSL protocol are shown in italics.

The geochemical and geochronological data therefore firmly establish the Urluia tephra as deriving from the CI super-eruption which occurred in the Phlegrean Fields of Italy.

### Nature of CI Tephra Deposition

The tephra deposit at Urluia consists of a distinct unit deposited on a gentle palaeoslope, increasing in thickness downslope (Figure 3a). It can be traced for more than 40 m along the quarry wall; colluvial deposits and inaccessible steep slopes resulting from quarrying activities obscure the primary stratigraphy along the rest of the quarry wall. At the thickest point near the depocentre of the palaeotopographic depression, the tephra reaches 100 cm thickness. At the southeastern end of the exposure, the tephra changes from a distinct, thick, fresh pale ash deposit approximately 15 cm in thickness to an intermittent exposure of blocks of more weathered orange-coloured tephra, the upper surface of which is orange in colour as a result of intensified in situ weathering. To the southeast, the tephra layer pinches out and can only be identified by gravel-sized orange-coloured clasts indicating its stratigraphic position. The distinction between the thicker, fresher tephra and thinner, more weathered deposits further upslope is most likely due to relatively prolonged exposure of the latter at the surface after deposition, and the higher vulnerability of thinner deposits to weathering. Although no screening has yet been performed, it is likely that the CI tephra might be present in cryptotephra form along the profile in sectors where the layer is not visible to the naked eye.

The tephra overlies pale, buff-coloured primary loess, and the contact between the two units is clearly defined (Figure 4b), suggesting a sudden transition. The contact between the tephra and overlying loess is more diffuse, indicating some degree of bioturbation and sediment mixing coeval with post-tephra loess deposition. The variation in thickness of this zone suggests the impact of overlying sedimentation on the comparatively less dense ash which created an irregular surface. At the northwest section, approximately mid-way along the palaeoslope, the tephra is ca. 50 cm thick and grades upwards from pure ash into a 25 cm thick zone comprising mixed tephra and loess. This layer is overlain by carbonate-rich loess, presumably representing HE4, which in turn is overlain by an interstadial weakly developed paleosol. This most intensely developed paleosol, containing humic components and carbonate rootlets, is ca. 1 m thick, and grades upwards through a transitional pedogenic zone into primary, light yellow loess a further 2.3 m above. This sequence may represent the Greenland Interstadials (GI) 8–5. The southeast section, where the sediments below the tephra were investigated, is interspersed with, and overlain by, weakly pedogenic overprinted loess (Figure 3b). The stratigraphic position, relatively weakly developed character and age of the overlying paleosol most likely correlates with the upper more intensively developed part of the L1S1 fossil soil complex identified in other loess profiles across the Danube basin [41], [42]. The L1S1 paleosols have been interpreted to correspond to relatively humid MIS 3 interstadial conditions prevailing not only across the Middle to Lower Danube basin [41] but also wider Eastern Europe [42], [67]. HE4 is situated within MIS 3 at the end of a series of relatively warm and humid interstadials characterizing early MIS 3 and prior to the Greenland Interstadials (GI) 8–5 representing the so-called Denekamp phase in the terrestrial palaeoclimate stratigraphy [14], [68].

The tephra is also clearly distinguished as a discrete stratigraphic unit within the loess by environmental magnetism, along with varying intensity of pedogenesis in the surrounding loess (Figure 3c). The tephra layer is sandwiched in between loess which shows incipient pedogensis and a weakly developed interstadial paleosol (maximum pedogenesis 0.5–1.0 m) above the tephra. The distinct peak in magnetic susceptibility can be visibly correlated with the overlying reworked tephra in the field; this layer yields χ-values almost one order of magnitude higher than the surrounding sediments. The weakly developed overlying interstadial paleosol can also be identified in the northwest section as a 2 m thick zone of generally increased magnetic susceptibility relative to the surrounding loess (Figure 3c; Table S5 in File S1); these χ-values are generally elevated and most likely mark the more favorable climate during MIS 3 (L1S1). The χ_fd_-values follow this trend but yield a distinct peak approximately 1 m below the tephra which cannot presently be explained. There is some indication of sediment redeposition during a phase of higher dust input between the tephra and interstadial paleosol which may correlate with HE4, although the resolution of sampling for environmental magnetism unfortunately precludes more detailed palaeoenvironmental reconstruction of this time period. There is no indication either sedimentologically or magnetically of more intense pedogenesis having taken place within the loess prior to tephra deposition.

A number of features indicate that the tephra was deposited rapidly at this site. At the contact between the tephra and underlying loess, imprints of vegetation are preserved (Figure 4d), along with intact ichnofossils of borings made by invertebrates as they attempted to escape the rapid deposition of ash. The low density of the tephra was sufficient not to distort or compact these trace fossils. The tephra itself is relatively poorly sorted, ranging from 10–250 µm diameter, with a high proportion of fine sand-sized clasts and a small component of grains >150 µm (Figure 4c). This grainsize range is broadly finer than the deposit at Crvena Stijena in Montenegro (500 km from the source; [53]) but certainly coarser than at Kostenki on the Russian Plain (2200 km distant from source; [8]). The Urluia site, more than 1200 km from the Phlegrean Fields, lies towards the hypothesized upper limit of distal sand-sized transport [53], [69], although without more detailed particle size analysis, further interpretations as to the magnitude of the eruption and associated suspended transport capacity of distal ash cannot be made.

Stratigraphic evidence at both micro and macro scales suggests minimal redeposition of the tephra at Urluia. Microlaminations and fine-scale cross-bedding are present within the unweathered tephra along the length of the exposure. While these features suggest some degree of transport during or just after the ashfall, the integrity of preservation of these sedimentary structures indicates that the bulk of this process occurred rapidly and prior to subsequent loess deposition. Likewise, the preservation of ichnofossils (traces of invertebrate burrowing and vegetation) strongly suggests rapid initial tephra deposition and minimal slumping over a short period of time.

In addition to previously documented exposures of the tephra on the Dobrogea Plateau and Danube Plain further north and west of the Urluia site [5], [31], several additional exposures nearby Urluia reinforce our arguments for its previously unrecognised thickness and ubiquity. In particular, an exposure on the Danube River at Rasova to the north of Urluia preserves a horizontal layer of sand-sized tephra up to 50 cm thick (Figure 1b), indicating another significant primary occurrence of CI tephra in the region. Another tephra layer is also exposed in a small road-cut profile on the other side of Urluia village approximately 1 km to the northeast. Although geochemical analyses are not yet available for this ash bed, its visual and microscopic optical appearance is identical to the Urluia tephra. It moreover occupies the same position within the loess-paleosol stratigraphy, suggesting that it derives from the same source. This latter ash bed is approximately 15 cm thick.

The stratigraphic and sedimentological evidence from the CI tephra deposit at Urluia, combined with emerging data from other deposits in the region ([5]; Figure 1b), strongly argue for widespread, thick, rapid sedimentation more than 1200 km from the source of the super-eruption.

## Discussion and Conclusions

### Implications for Reconstructing the Scale of the Eruption

The evidence from our study indicates rapid deposition of coarse-grained, distal tephra from the CI super-eruption more than 1200 km from its source.

The unusually thick deposit of CI tephra at Urluia Quarry in the Dobrogea region of southeastern Romania is at its thickest point up to twenty times thicker than is proposed from the computational model for this region (Figure 1; [12]). In addition, the exposure at Urluia corroborates recent observations of 12 additional substantial accumulations across the lower Danube basin (Figure 1b), of which at least four [5], described in detail, are up to five times thicker than proposed by the model. In our view, these data provide a critical mass of information sufficient to alter the computational models for the distal transport of tephra, and will most likely increase the volume of ash and magnitude of the eruption substantially. Accordingly, the climatic impacts of the CI super-eruption, its interaction with the HE4 episode, and implications for Palaeolithic communities living in this region especially, are likely to have been more extreme, and should be carefully reassessed.

### Implications for Palaeoclimate and Human Evolution

There is some indication from both the stratigraphic and environmental magnetic profile at Urluia that the CI tephra deposition was immediately followed by a short-lived phase of loess deposition associated with stadial conditions, which is overlain by the interstadial paleosol (Figure 3c). This phase may be correlated with HE4, although at present the resolution of the record is insufficient to extract the intensity of this climate signal. However, if this interpretation is accurate, then it corroborates studies from southern Italy which place the timing of the CI super-eruption at the onset of HE4 [32], [33]. Primary loess deposition at Urluia would indicate cooler, more arid conditions, which have been noted in records from central and western Europe at this time [32], [37], [38], but are as yet poorly defined in eastern Europe [41], [42]. At present, however, the potential feedback link between the CI volcanic event and HE4 cannot be established beyond the hypothetical realm [11]. However, since most of southeastern and eastern Europe lies on the “fringe area” of the eruption’s impact, should a positive feedback cycle have been established, the region, and the hominins and fauna living within it, would certainly have experienced the climatic deterioration caused by the coupling of HE4 with a volcanic winter [48].

The potential effects of the CI super-eruption and related ashfall on hominin populations are manifold. Our stratigraphic data from the more distal parts of Eastern Europe, combined with a now critical mass of additional tephra occurrences in various sedimentary settings [5], [6], [70]–[72], suggest that even such distal regions may have experienced direct and substantial effects of ash deposition. Among the known health hazards associated with volcanic ashes are a variety of respiratory acute (e.g., asthma) and chronic diseases (e.g., silicosis) caused by the inhalation of fine ash particles [73]. The magnitude of these effects is directly related to the proportion of finer, respirable ash grains, implying that areas situated at different distances from the eruption should experience different localized effects, despite similar ashfall volumes [74]. A further effect of substantial ashfall is fluoride poisoning, which may affect not only people [75] but also fauna, particularly large herbivores [55]. Both modern [76] and archaeological [77] examples of fluoride poisoning have been documented. Fluorosis can lead to death, but it is the associated longer term debilitating bone deformations (e.g., hyperostosis, osteosclerosis, osteomalacia, and osteoporosis) that are most likely to be identifiable in the archaeological record [77].

Archaeological traces of the response of hunter-gatherer populations to the effects of volcanic eruptions have been studied in a variety of contexts [74], [78]–[84], and the patterns differ markedly from case to case. The Toba super-eruption in Sumatra (ca. 74 ka; [46]) seems to have induced few changes in hunter-gatherer adaptations in, for example, the distal Jurreru Valley in India [82], [83], even though ashfall there was also substantial. However, despite being one of the largest volcanic events during the late Pleistocene, Toba is perhaps not the most appropriate analogy for the effects of the CI super-eruption [48], since the equatorial location of the eruption result in tephra predominantly falling over the ocean would have induced different environmental impacts [48], [85]; furthermore, the temporal resolution of the archaeological record there is low [48]. More relevant to our case is the Laacher See eruption in the Eifel region of Germany, an intraplate volcanic event which occurred at temperate latitudes ca. 12.9 ka ago, approximately two centuries prior to the Younger Dryas abrupt cooling event [86]. The Laacher See eruption appears to correlate with major technological changes, such as the abandonment of the bow and arrow [87]. Technological changes can also result from adaptation to decreased subsistence yields in the environment [88], which may be independent of volcanic eruptions but is nevertheless predicted, possibly on a continental scale, by the magnitude of the combined impact of the CI-HE4 event.

Given the above-mentioned potential effects of super-eruptions on hominin populations, what is the evidence we currently see on the ground? A recent study of several CI-tephra-bearing archaeological sites in the southern and central Balkans interpreted an absence of evidence for a catastrophic effect on human populations, in the form of discontinuities, as evidence for the resilience of populations in the region and the survival of cultural traditions [10]. This contrasts with archaeological sites proximal to the eruption in southern Italy which show a marked hiatus in occupation [3], [49], [50], with the lithic artifact record at Crvena Stijena in Montenegro east of the Adriatic Sea which suggests that the CI tephra coincides with the boundary between Middle and Upper Palaeolithic technologies [53], and with the Temnata Cave record in Bulgaria which also suggests a transition in lithic industries [70]. The currently known distribution of the thickest ash layers also suggest that it is possible that local population histories may vary even across a small region. Ultimately, however, the region presently lacks systematic and concrete diachronic studies over large areas where the effects of the CI super-eruption on hominin populations can be properly tested. Consequently, we will refrain from speculation regarding the presence or absence of specific hominin species in the region at the time of the eruption, except to say that current data suggest a patchwork structure. The difficulty in pronouncing a judgment on this issue arises mainly from the fact that the number of known archaeological sites in Eastern Europe securely dated to this period is so few that an accurate view of paleodemography is not yet possible. Nevertheless it is likely that Eastern Europe, and particularly the Danube Basin, was at this time a crossroads between migration routes of AMHs from the south and possibly from the east across the northern Pontic area [56], [89], producing a cultural mosaic that is difficult to decipher today. This is further complicated by the fact that most of the landscape under consideration is draped by thick loess and alluvial deposits that might obscure traces of past human presence [41], [90]. Nevertheless, AMHs were clearly already present in the Carpathian region at, or slightly before, the time of the CI super-eruption, as demonstrated by the Peştera cu Oase AMH fossil remains [91]. However, because these fossils are not associated with any archaeology, rendering a direct association between stone tools and hominin morphology is currently impossible. In practice, this means that migration routes are proposed by establishing at best tenuous cultural-historical links between local lithic assemblages and more geographically distant techno-complexes (e.g., the Near-Eastern Ahmarian or Emiran) that are more securely associated with hominin species (i.e. AMHs, Neandertals). The process is not helped by complex, locally-defined lithic typologies, assumptions of unilinear progress in time, and, not least, by the frequent use of quartzite or other coarse-grained raw materials which render the typological approach difficult or inconclusive [92].

Speaking probabilistically, focusing on long archaeological sequences (usually in caves or rockshelters) in regions distal to the super-eruption represents our best chance of sampling the time interval of interest. At present, the discrepancy between the archaeological data offered by the few cave sequences in the southern and central Balkans (e.g. Klissoura, Golema Pesht, Franchthi, and Tabula Traiana; [10]), the tephra-bearing long rockshelter sequence at Crvena Stijena [53], and the open-air non-archaeological situations documented in Dobrogea (this study) and southern Romania [5] calls for a more intense and systematic investigation of open-air archives in the region. So far, only a cluster of recently re-excavated archaeological sites in the Romanian Banat (Româneşti-Dumbrăviţa/Coşava/Tincova; [93]) and the Petrovaradin Fortress site in Vojvodina (northern Serbia; [94]) appear to contain the relevant time intervals, but have not yet yielded information regarding tephra deposits, nor is the dating sufficiently precise to be sure that they were occupied at the precise time of the CI super-eruption. Recent systematic survey in Dobrogea has so far documented and dated a number of Palaeolithic sites, constrained to the later Middle Palaeolithic (Cuza Vodă; [95]) and last glacial period [96], but so far no sites have been identified that date to the CI super-eruption or HE4. Given existing data, it remains difficult to evaluate the impact of, and response to, the eruption in Eastern Europe. However, given the high visibility and widespread distribution of the CI tephra across the region, intensive, targeted surveys, making use of modern subsurface surveying techniques, should uncover such sites if they exist. Efforts within our own research programme are currently focused on finding new archaeological sites associated with CI tephra deposits in Southeastern Romania [95].

In conclusion, we have demonstrated that CI ash deposits in the Lower Danube steppe are much thicker than previously modelled. Moreover, the ashfall present at Urluia was deposited quickly and might have constituted a health hazard for mammalian taxa inhabiting the region, including hominins, irrespective of species. Although we have at present no archaeological remains associated with the deposit, the visibility of the tephra in several profiles in the region is encouraging for targeted surveys.

## Materials and Methods

The profile at Urluia was cleaned and logged at three sections along the palaeotopographic slope, to cover the range of tephra thickness laterally along the outcrop. The contact between the tephra and the loess immediately underlying and overlying it was also observed with respect to documenting the associated sedimentary features and soil development. Two main sections (“northwest” and “southeast”) were selected for detailed investigation (Figure 3b). The tephra layer at the northwest profile is of intermediate thickness (ca. 50 cm) but was more accessible for sampling than the thickest (100 cm) exposure of the tephra. Two samples were collected for geochemical analysis from the base and top of the tephra within the northwestern cleaned section. Block samples of the tephra were also collected from the lower contact with the loess to observe the nature of deposition. Sediment samples for environmental magnetism studies were collected both from the northwest (72 samples) and southeast (55 samples) cleaned sections. Luminescence dating samples were collected in stainless steel, lightproof tubes driven horizontally into the section above and below the tephra at the northwest (3 samples) and southeast (2 samples) cleaned sections. In total, three samples were collected from below the tephra, and two samples were collected from above it. Additional bulk sediment samples were collected from immediately surrounding the tube locations for laboratory analysis of dosimetry.

### Geochemistry

Two samples were collected for geochemical analyses from the northwest section, from the base (URL1) and top (URL2) of the tephra. The ash samples were prepared in the laboratory using published protocols [5]. The almost pure tephra was disaggregated by gently pressing the material and then mounted in epoxy resin, ground and polished in preparation for microprobe analysis of 10 major and minor element concentrations. Measurements were made using single-grain, wavelength-dispersive electron microprobe analysis [5] on up to 40 grains from each sample. The measurements were performed at the Bayerisches GeoInstitut (University of Bayreuth) on a Jeol JXA8200 microprobe employing an accelerating voltage of 15 keV. A 6 nA beam current and defocussed beam were used. Peak counting times were 10 s for Na, 30 s for Si, Al, K, Ca, Fe and Mg, 40 s for Ti and Mn, and 60 s for P. Precision is estimated at <1–6% (2σ) and 10–25% (2σ) for major and minor element concentrations respectively.

### Geochronology

The five luminescence dating samples were processed in the laboratory using published protocols to extract fine-grained (4–11 µm) polymineral material [97]. A subsample of this material was then etched in hydrofluorosilicic acid to extract fine-grained quartz following published laboratory procedures [98]; three of the five samples prepared with this procedure yielded sufficient quartz extract for equivalent dose measurements. Equivalent dose estimation was undertaken on each sample using 24 aliquots on a Risø TL-DA-20 reader [99], [100], using a U340 filter for quartz OSL measurements, and a D410 filter for polymineral post-IR IRSL measurements. Polymineral samples were measured using the post-IR IRSL_290_ protocol [101]. The fine-grained quartz samples were measured using the single aliquot regenerative dose (SAR) protocol [102] using a preheat temperature of 240°C, based on the results of a preheat plateau test for thermal stability on a loess sample overlying the tephra (Figure S1 in File S1). Dose recovery (Figure S2 in File S1 and Table S2 in File S1) and recycling ratios (Table S3 in File S1) lay within 5% and 10% of unity respectively, indicating suitability for dating. Since the resulting dose distributions arising from each series of aliquots yielded Gaussian populations (Figure S3 in File S1), the equivalent dose for each sample was determined using the central age model (CAM) [103]. Dose rates for the beta and gamma components were derived from high resolution germanium gamma spectrometry measured at the Felsenkeller in Dresden, Germany, compared with in-house beta counting, and corrected using published attenuation factors [104] incorporating the averaged moisture contents from the sample tubes and surrounding bulk sediment samples (Table S4 in File S1). Alpha values of 0.04±0.02 and 0.08±0.02 were used for the quartz and polymineral fine grain samples respectively to account for the lower luminescence efficiency of alpha radiation relative to the beta and gamma components [105], [106]. Cosmic dose rates were calculated using published formulae [107]. Further data relating to luminescence dating characteristics can be found in the Supplementary Information section (File S1).

### Environmental Magnetism

Environmental magnetism in loessic sediments is applied on the principle enhancement of magnetic minerals derived from silicate weathering, primarily iron oxides and hydroxides, through pedogenesis [108], [109], and in this case, also the presence of weathered and fresh volcanic glass and minerals. Since pedogenesis is climatically controlled, variations in magnetic susceptibility down profile can be linked to regional climatic changes such as glacial-interglacial variations, or if the resolution allows, even to stadial-interstadial fluctuations.

Samples for magnetic susceptibility analyses were taken both from the southeast profile (55 samples) and northwest profile (72 samples), from cleaned sections at 5 cm intervals (Table S5 in File S1). Sampling at the northwest profile focused on the loess-palaeosol sequence overlying the tephra, including the contact zone of mixed loess and tephra. At the southeast profile, samples were collected from the primary (L1) loess underlying the tephra. Stratigraphically, the sections are in contact with the tephra as a reference horizon. Samples were collected in air-tight plastic bags and dried at 40°C in the laboratory, then packed in 6.4 cm^3^ plastic boxes. Magnetic susceptibility measurements were undertaken using an AGICO KLY-3 3-Spinner-Kappa-Bridge reader (AGICO, Brno, Czech Republic) working at 920 Hz and 300 A m^−1^, using previously published methods [67], [110]. For measurement of the frequency-dependent magnetic susceptibility (χ_fd_), each specimen was measured twice at two different frequencies (0.3 and 3 kHz; χ@_0.3kHz_ - χ@_3kHz_/χ@_0.3kHz_*100 in %) in a magnetic AC field of 300 Am^−1^ using a MAGNON VFSM susceptibility bridge (MAGNON, Dassel, Germany). The data are expressed as mass specific magnetic susceptibility (χ in kg^−1^ m^3^) and frequency-dependent magnetic susceptibility (χ_fd_ in %), and used as first order proxies for identifying the course of the intensity of pedogenesis with depth within the stratigraphy.

The measurement of χ_fd_ is sensitive to the presence and relative contribution of ultrafine (c. ≤30 nm), pedogenetically derived, so called superparamagnetic (SP) particles in a sample. For that reason this parameter is often used as a proxy for pedogenetic formed SP particles and thus for pedogenesis [111].

## Supporting Information

File S1 **This file contains supporting figures and tables.** Table S1, Geochemical analyses of tephra samples URL1 and URL2. Grains analysed yielding total % concentrations of <95% have been removed from the dataset. Table S2, Results of dose recovery tests for quartz OSL samples L-EVA1089 and L-EVA1090. Both samples were given a calibrated known dose of 42.75 Gy. Table S3, Averaged measured recycling ratios and standard deviations measured for each luminescence dating sample. Samples measured using quartz OSL are shown in plain text; those measured using the post-IR IRSL_290_ protocol are given in italics. Table S4, Moisture contents (and incorporated uncertainties) of the luminescence dating samples. Table S5, Mass specific and frequency dependent magnetic susceptibility of samples from subsections SS1 (NW profile) and SS2 (SE profile). Susceptibilities were measured in a field of 300 Am^−1^ at frequencies of 300 and 3000 Hz, respectively. Figure S1, Results of preheat plateau test for sample L-EVA1027. Figure S2, Recovered dose distributions from dose recovery tests on samples L-EVA1089 and L-EVA1090. Figure S3, Radial plots illustrating the dose distributions of each of the luminescence dating samples.
(DOCX)
