# Supplementary material for: The Campanian Ignimbrite Eruption: New Data on Volcanic Ash Dispersal and Its Potential Impact on Human Evolution
Source: PLoS One. 2013 Jun 17;8(6):e65839. doi: 10.1371/journal.pone.0065839 (PMC3684589; doi:10.1371/journal.pone.0065839)
Supplement: File S1 — This file contains supporting figures and tables. Table S1, Geochemical analyses of tephra samples URL1 and URL2. Grains analysed yielding total % concentrations of <95% have been removed from the dataset. Table S2, Results of dose recovery tests for quartz OSL samples L-EVA1089 and L-EVA1090. Both samples were given a calibrated known dose of 42.75 Gy. Table S3, Averaged measured recycling ratios and standard deviations measured for each luminescence dating sample. Samples measured using quartz OSL are shown in plain text; those measured using the post-IR IRSL290 protocol are given in italics. Table S4, Moisture contents (and incorporated uncertainties) of the luminescence dating samples. Table S5, Mass specific and frequency dependent magnetic susceptibility of samples from subsections SS1 (NW profile) and SS2 (SE profile). Susceptibilities were measured in a field of 300 Am−1 at frequencies of 300 and 3000 Hz, respectively. Figure S1, Results of preheat plateau test for sample L-EVA1027. Figure S2, Recovered dose distributions from dose recovery tests on samples L-EVA1089 and L-EVA1090. Figure S3, Radial plots illustrating the dose distributions of each of the luminescence dating samples. (DOCX) [file pone.0065839.s001.docx]

**Supplementary Information**

**Geochemical analyses**

**Table S1.** Geochemical analyses of tephra samples URL1 and URL2. Grains analysed yielding total % concentrations of <95% have been removed from the dataset.

| **Grain** | **Concentrations** | | | | | | | | | | | |
| --- | --- | --- | --- | --- | --- | --- | --- | --- | --- | --- | --- | --- |
|  | **Na_2_O** | **SiO_2_** | **K_2_O** | **CaO** | **FeO** | **MgO** | **Al_2_O_3_** | **P_2_O_5_** | **TiO_2_** | **MnO** | **Cl** | **Total** |
| ***URL1*** | | | | | | | | | | | | |
| 1 | 6.58 | 59.71 | 6.61 | 1.60 | 2.87 | 0.34 | 18.84 | 0.02 | 0.40 | 0.24 | 0.79 | 98.00 |
| 2 | 6.43 | 58.50 | 6.63 | 1.62 | 2.89 | 0.32 | 18.17 | 0.01 | 0.40 | 0.23 | 0.72 | 95.92 |
| 3 | 6.01 | 59.19 | 6.97 | 1.65 | 2.76 | 0.32 | 18.30 | 0.03 | 0.39 | 0.23 | 0.69 | 96.54 |
| 4 | 6.42 | 59.52 | 6.76 | 1.66 | 2.72 | 0.35 | 18.47 | 0.01 | 0.40 | 0.21 | 0.68 | 97.21 |
| 5 | 6.55 | 60.15 | 6.67 | 1.71 | 2.87 | 0.29 | 18.67 | 0.04 | 0.43 | 0.26 | 0.77 | 98.41 |
| 6 | 6.53 | 60.32 | 7.03 | 1.66 | 2.80 | 0.33 | 18.72 | 0.05 | 0.39 | 0.25 | 0.71 | 98.80 |
| 7 | 6.24 | 59.25 | 6.77 | 1.63 | 2.79 | 0.34 | 18.19 | 0.02 | 0.41 | 0.24 | 0.75 | 96.62 |
| 8 | 6.14 | 58.77 | 6.88 | 1.70 | 2.79 | 0.39 | 18.16 | 0.04 | 0.41 | 0.27 | 0.78 | 96.34 |
| 9 | 6.72 | 60.80 | 7.05 | 1.67 | 2.86 | 0.39 | 18.62 | 0.04 | 0.43 | 0.24 | 0.73 | 99.54 |
| 10 | 6.72 | 61.00 | 6.67 | 1.68 | 2.91 | 0.30 | 18.85 | 0.06 | 0.41 | 0.23 | 0.73 | 99.56 |
| 11 | 6.35 | 60.27 | 6.77 | 1.62 | 2.74 | 0.34 | 18.50 | 0.07 | 0.47 | 0.24 | 0.75 | 98.11 |
| 12 | 6.24 | 59.49 | 6.72 | 1.77 | 2.75 | 0.34 | 18.41 | 0.01 | 0.41 | 0.20 | 0.76 | 97.10 |
| 13 | 5.60 | 58.92 | 7.52 | 1.70 | 2.88 | 0.32 | 18.41 | 0.05 | 0.40 | 0.21 | 0.77 | 96.79 |
| 14 | 3.00 | 57.72 | 9.26 | 2.62 | 3.30 | 0.77 | 17.79 | 0.15 | 0.38 | 0.13 | 0.28 | 95.39 |
| 15 | 3.73 | 59.85 | 8.95 | 2.35 | 3.13 | 0.66 | 17.97 | 0.19 | 0.33 | 0.07 | 0.35 | 97.58 |
| 16 | 4.14 | 61.27 | 8.74 | 2.62 | 3.22 | 0.73 | 18.36 | 0.12 | 0.35 | 0.12 | 0.33 | 99.99 |
| 17 | 6.16 | 59.51 | 6.91 | 1.69 | 2.84 | 0.33 | 18.57 | 0.05 | 0.44 | 0.23 | 0.72 | 97.44 |
| 18 | 6.59 | 61.54 | 7.15 | 1.74 | 2.92 | 0.39 | 18.64 | 0.02 | 0.40 | 0.23 | 0.72 | 100.34 |
| 19 | 6.83 | 61.23 | 7.06 | 1.72 | 2.85 | 0.35 | 18.68 | 0.03 | 0.40 | 0.19 | 0.78 | 100.13 |
| 20 | 6.77 | 60.81 | 6.86 | 1.67 | 2.88 | 0.35 | 18.66 | 0.00 | 0.50 | 0.20 | 0.69 | 99.40 |
| 21 | 6.45 | 59.41 | 6.64 | 1.59 | 2.88 | 0.31 | 18.29 | 0.03 | 0.43 | 0.23 | 0.70 | 96.96 |
| 22 | 5.01 | 61.01 | 7.58 | 1.81 | 2.67 | 0.35 | 18.04 | 0.04 | 0.38 | 0.14 | 0.54 | 97.57 |
| 23 | 6.28 | 60.04 | 6.79 | 1.68 | 2.82 | 0.35 | 18.70 | 0.05 | 0.39 | 0.21 | 0.66 | 97.97 |
| 24 | 6.89 | 61.26 | 7.16 | 1.77 | 2.91 | 0.32 | 18.94 | 0.06 | 0.60 | 0.25 | 0.76 | 100.81 |
| 25 | 5.90 | 59.54 | 6.80 | 1.62 | 2.69 | 0.33 | 18.61 | 0.04 | 0.36 | 0.22 | 0.76 | 96.86 |
| 26 | 5.90 | 59.85 | 7.01 | 1.71 | 2.74 | 0.35 | 18.51 | 0.02 | 0.42 | 0.22 | 0.65 | 97.16 |
| 27 | 5.18 | 57.78 | 7.81 | 2.05 | 2.85 | 0.39 | 18.86 | 0.00 | 0.42 | 0.18 | 0.75 | 96.25 |
| 28 | 6.01 | 60.18 | 7.16 | 1.64 | 2.80 | 0.36 | 18.52 | 0.05 | 0.39 | 0.23 | 0.67 | 98.10 |
| 29 | 5.94 | 58.45 | 6.92 | 1.75 | 2.79 | 0.34 | 18.10 | 0.05 | 0.46 | 0.21 | 0.73 | 95.74 |
| 30 | 6.88 | 61.31 | 7.24 | 1.76 | 2.91 | 0.32 | 18.68 | 0.05 | 0.44 | 0.21 | 0.75 | 100.54 |
| 31 | 6.60 | 59.43 | 6.75 | 1.68 | 2.84 | 0.37 | 18.36 | 0.04 | 0.37 | 0.22 | 0.75 | 97.42 |
| 32 | 6.69 | 60.72 | 7.02 | 1.76 | 2.87 | 0.40 | 18.71 | 0.05 | 0.44 | 0.24 | 0.73 | 99.63 |
| 33 | 6.63 | 59.53 | 6.67 | 1.65 | 3.01 | 0.39 | 18.43 | 0.07 | 0.46 | 0.26 | 0.73 | 97.82 |
| 34 | 4.70 | 59.86 | 7.45 | 2.07 | 2.63 | 0.46 | 18.10 | 0.09 | 0.34 | 0.17 | 0.43 | 96.30 |
| 35 | 6.25 | 58.86 | 6.68 | 1.66 | 2.76 | 0.35 | 18.40 | 0.02 | 0.44 | 0.23 | 0.71 | 96.36 |
| 36 | 6.72 | 61.38 | 6.88 | 1.81 | 2.91 | 0.36 | 18.76 | 0.03 | 0.47 | 0.20 | 0.76 | 100.28 |
| 37 | 6.41 | 59.90 | 7.10 | 1.63 | 2.82 | 0.36 | 18.78 | 0.01 | 0.42 | 0.24 | 0.73 | 98.39 |
| 39 | 6.53 | 61.02 | 6.82 | 1.65 | 2.94 | 0.32 | 18.80 | 0.06 | 0.43 | 0.20 | 0.77 | 99.54 |
| 40 | 6.65 | 60.56 | 6.97 | 1.63 | 2.83 | 0.34 | 18.83 | 0.00 | 0.40 | 0.23 | 0.80 | 99.23 |
| *Average* | *6.08* | *59.95* | *7.10* | *1.77* | *2.86* | *0.38* | *18.48* | *0.05* | *0.41* | *0.21* | *0.69* |  |
| *S.D.* | *0.87* | *0.99* | *0.61* | *0.24* | *0.13* | *0.10* | *0.21* | *0.04* | *0.04* | *0.04* | *0.13* |  |
| ***URL2*** | | | | | | | | | | | | |
| 1 | 4.03 | 59.51 | 8.55 | 2.06 | 2.88 | 0.54 | 18.09 | 0.09 | 0.42 | 0.09 | 0.38 | 96.64 |
| 2 | 4.97 | 61.35 | 8.22 | 1.82 | 2.70 | 0.45 | 18.37 | 0.07 | 0.33 | 0.14 | 0.42 | 98.83 |
| 3 | 3.94 | 64.96 | 10.59 | 0.71 | 0.25 | 0.01 | 19.37 | 0.00 | 0.01 | 0.00 | 0.03 | 99.87 |
| 5 | 5.81 | 61.92 | 7.52 | 1.97 | 2.80 | 0.42 | 18.44 | 0.08 | 0.36 | 0.15 | 0.49 | 99.96 |
| 6 | 6.54 | 60.21 | 7.12 | 1.68 | 2.92 | 0.32 | 18.68 | 0.03 | 0.43 | 0.21 | 0.74 | 98.88 |
| 8 | 4.97 | 57.78 | 8.02 | 2.33 | 3.36 | 0.66 | 18.78 | 0.15 | 0.37 | 0.18 | 0.59 | 97.19 |
| 9 | 5.15 | 60.93 | 7.25 | 2.76 | 3.43 | 0.80 | 18.68 | 0.18 | 0.36 | 0.08 | 0.36 | 99.98 |
| 10 | 5.73 | 59.02 | 6.81 | 1.71 | 2.86 | 0.35 | 18.44 | 0.08 | 0.41 | 0.24 | 0.73 | 96.38 |
| 11 | 6.21 | 60.51 | 7.25 | 1.73 | 2.92 | 0.36 | 18.84 | 0.04 | 0.40 | 0.22 | 0.71 | 99.20 |
| 12 | 6.70 | 60.12 | 7.07 | 1.67 | 2.88 | 0.36 | 18.78 | 0.07 | 0.41 | 0.23 | 0.78 | 99.08 |
| 13 | 3.18 | 59.76 | 9.85 | 2.57 | 3.34 | 0.75 | 18.25 | 0.19 | 0.36 | 0.12 | 0.27 | 98.63 |
| 14 | 6.06 | 58.74 | 6.63 | 1.69 | 2.80 | 0.32 | 18.29 | 0.07 | 0.34 | 0.22 | 0.74 | 95.91 |
| 15 | 6.62 | 59.55 | 6.68 | 1.74 | 2.91 | 0.34 | 18.38 | 0.07 | 0.42 | 0.25 | 0.75 | 97.71 |
| 16 | 4.95 | 59.95 | 7.08 | 1.88 | 2.72 | 0.41 | 18.00 | 0.07 | 0.32 | 0.13 | 0.40 | 95.91 |
| 17 | 6.53 | 59.52 | 7.31 | 1.79 | 2.86 | 0.33 | 18.63 | 0.03 | 0.40 | 0.24 | 0.70 | 98.35 |
| 18 | 3.26 | 59.07 | 9.49 | 2.45 | 3.43 | 0.75 | 17.97 | 0.18 | 0.38 | 0.09 | 0.29 | 97.35 |
| 19 | 6.56 | 60.49 | 7.15 | 1.79 | 2.98 | 0.32 | 18.58 | 0.04 | 0.42 | 0.23 | 0.73 | 99.29 |
| 20 | 6.76 | 60.83 | 7.11 | 1.76 | 2.90 | 0.36 | 18.85 | 0.02 | 0.41 | 0.24 | 0.76 | 99.99 |
| 21 | 3.05 | 59.46 | 9.86 | 2.52 | 3.29 | 0.75 | 18.16 | 0.15 | 0.36 | 0.08 | 0.31 | 97.99 |
| 22 | 5.57 | 62.23 | 7.39 | 1.79 | 2.77 | 0.37 | 18.77 | 0.06 | 0.36 | 0.19 | 0.56 | 100.06 |
| 25 | 3.74 | 59.38 | 9.08 | 2.22 | 2.96 | 0.55 | 18.09 | 0.14 | 0.37 | 0.10 | 0.41 | 97.04 |
| 27 | 6.57 | 58.68 | 6.73 | 1.64 | 2.84 | 0.35 | 18.31 | 0.06 | 0.41 | 0.22 | 0.77 | 96.58 |
| 28 | 6.08 | 58.72 | 7.18 | 1.79 | 2.83 | 0.30 | 18.34 | 0.02 | 0.38 | 0.21 | 0.73 | 96.58 |
| 29 | 5.31 | 61.79 | 7.68 | 2.01 | 2.74 | 0.45 | 18.50 | 0.05 | 0.38 | 0.12 | 0.47 | 99.50 |
| 30 | 4.35 | 60.51 | 8.97 | 2.40 | 3.34 | 0.67 | 18.49 | 0.18 | 0.34 | 0.16 | 0.33 | 99.73 |
| 31 | 6.26 | 58.92 | 6.89 | 1.66 | 3.01 | 0.31 | 18.42 | 0.03 | 0.43 | 0.22 | 0.74 | 96.89 |
| 32 | 6.64 | 58.56 | 6.72 | 1.72 | 2.94 | 0.31 | 18.44 | 0.04 | 0.39 | 0.23 | 0.73 | 96.71 |
| 33 | 6.56 | 59.73 | 6.82 | 1.69 | 2.91 | 0.37 | 18.26 | 0.05 | 0.39 | 0.23 | 0.69 | 97.70 |
| 35 | 6.53 | 58.39 | 7.42 | 1.73 | 2.78 | 0.36 | 18.04 | 0.01 | 0.38 | 0.21 | 0.76 | 96.62 |
| 36 | 6.49 | 59.50 | 6.78 | 1.69 | 2.88 | 0.37 | 18.51 | 0.09 | 0.39 | 0.24 | 0.73 | 97.67 |
| 37 | 6.28 | 59.34 | 7.02 | 1.72 | 2.90 | 0.34 | 18.51 | 0.05 | 0.39 | 0.24 | 0.71 | 97.50 |
| 39 | 3.78 | 59.79 | 8.83 | 2.51 | 3.28 | 0.68 | 18.28 | 0.16 | 0.35 | 0.10 | 0.33 | 98.09 |
| 40 | 6.81 | 59.99 | 6.87 | 1.77 | 2.94 | 0.35 | 18.76 | 0.00 | 0.46 | 0.23 | 0.74 | 98.91 |
| *Average* | *5.24* | *58.76* | *7.53* | *1.83* | *2.75* | *0.42* | *18.61* | *0.09* | *0.36* | *0.17* | *0.54* |  |
| *S.D.* | *1.56* | *3.87* | *2.04* | *0.47* | *0.73* | *0.19* | *2.65* | *0.06* | *0.10* | *0.07* | *0.23* |  |

**Luminescence dating**

This section contains additional data relevant to the luminescence dating component of this study, including information pertaining to luminescence characteristics and radial plots for the single grain measurements on each sample.

As described in the main text, equivalent dose (D_e_) estimation was undertaken on 24 aliquots each using either the single aliquot regenerative dose (SAR) protocol on fine-grained quartz (L-EVA1028, 1089, 1090) or the post-IR IRSL_290_ protocol on polymineral fine-grained samples for which insufficient quartz was recovered for measurement (L-EVA1029, 1091). Prior to sample measurement, a preheat plateau test for thermal stability was undertaken on a loess sample overlying the tephra (L-EVA1027); based on these results (Figure S1), a preheat temperature of 240˚C was selected for the quartz OSL measurements.

**Figure S1.** Results of preheat plateau test for sample L-EVA1027.


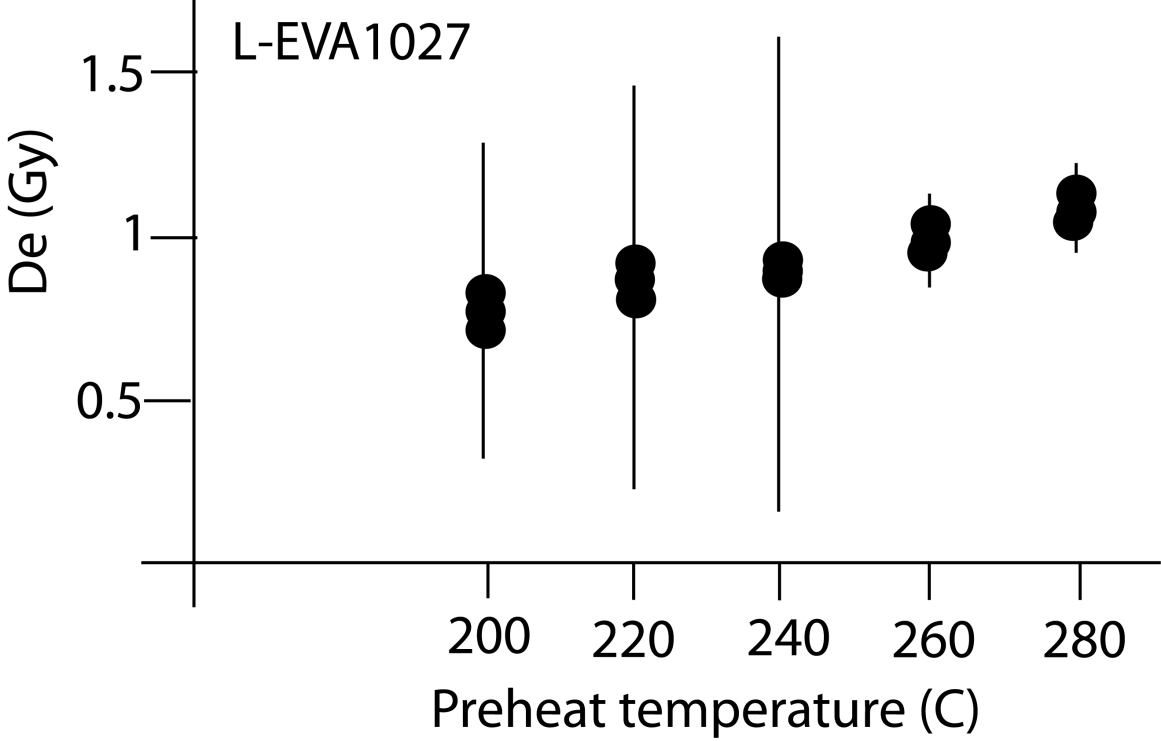


Dose recovery tests were undertaken on two samples bracketing the tephra (L-EVA1089, 1090) to investigate their suitability for dating based on the recovery of a known radiation dose (42.75 Gy). Residual signal within both samples was removed by laboratory bleaching with blue-green LEDs for 300 s. Both quartz OSL samples yielded dose recovery ratios within 5% of unity (Table S2; Figure S2), and consequently are interpreted as suitable for luminescence dating.

**Table S2.** Results of dose recovery tests for quartz OSL samples L-EVA1089 and L-EVA1090. Both samples were given a calibrated known dose of 42.75 Gy.

| **Sample** | **Recovered D_e_ (Gy) ± standard error** | **σ (%)** | **Dose recovery ratio** |
| --- | --- | --- | --- |
| L-EVA1089 | 44.5 ± 1.2 | 0.0 | 1.04 |
| L-EVA1090 | 41.6 ± 1.2 | 1.4 | 0.97 |

**Figure S2.** Recovered dose distributions from dose recovery tests on samples L-EVA1089 and L-EVA1090.


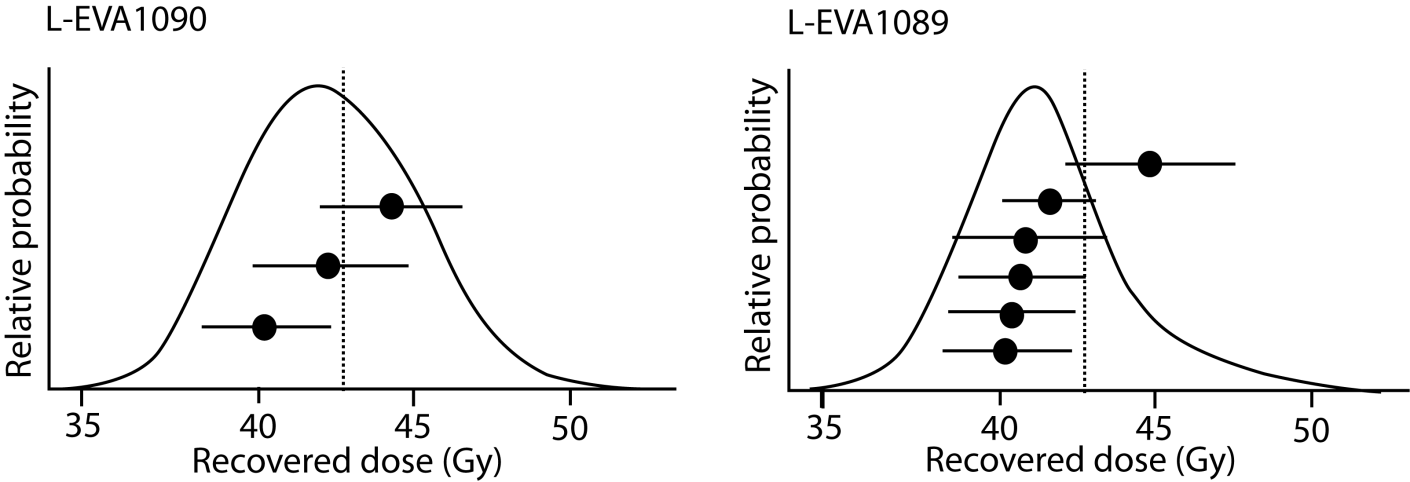


The dose distributions arising from each series of aliquots are shown below. The recycling ratios of each sample are given in Table S3. Samples measured using quartz OSL are shown in plain text; those measured using the post-IR IRSL_290_ protocol are given in italics.

**Figure S3.** Radial plots illustrating the dose distributions of each of the luminescence dating samples.

**
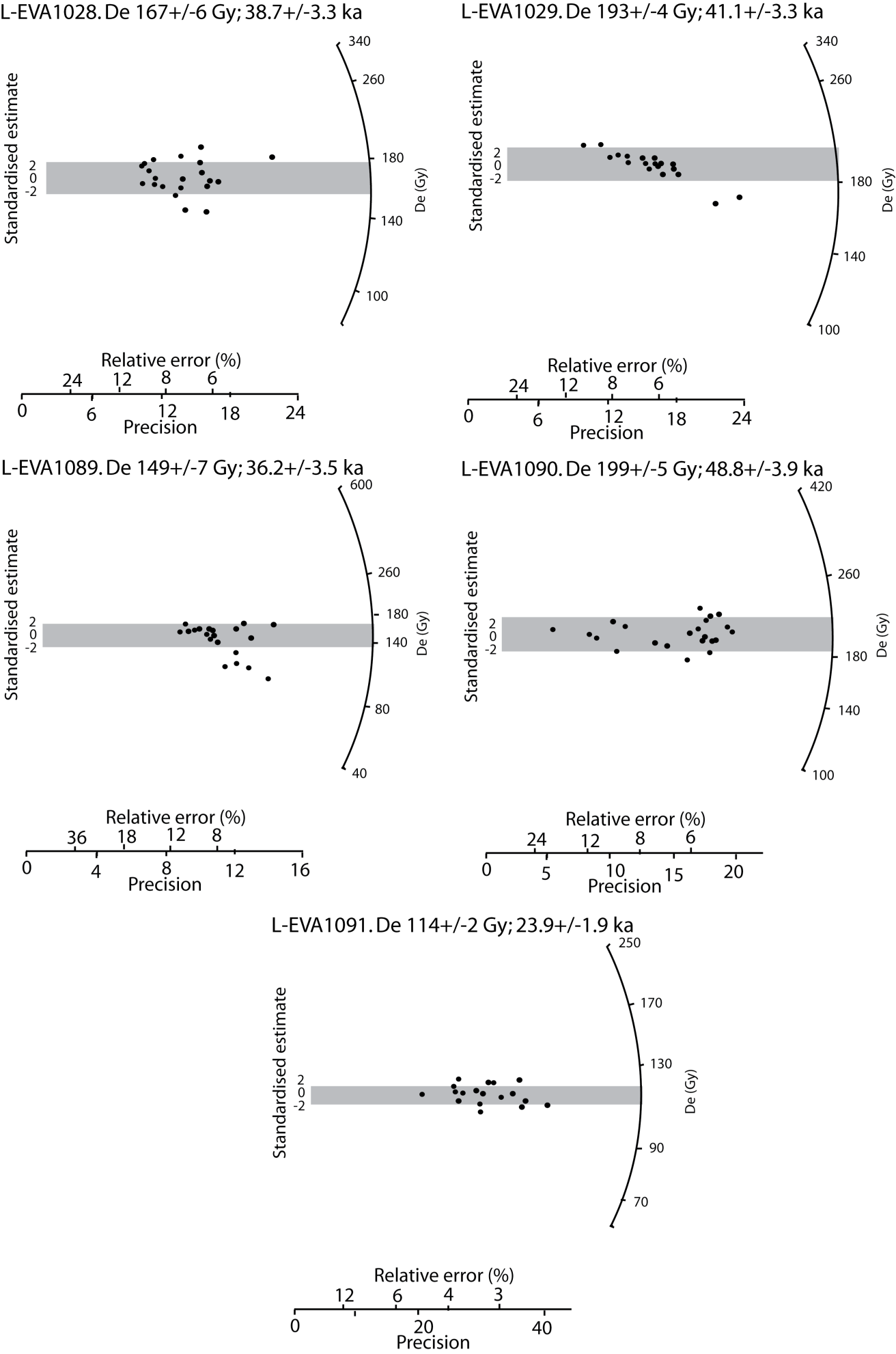
**

**Table S3.** Averaged measured recycling ratios and standard deviations measured for each luminescence dating sample. Samples measured using quartz OSL are shown in plain text; those measured using the post-IR IRSL_290_ protocol are given in italics.

| **Sample** | **Recycling ratio ± S.D.** |
| --- | --- |
| L-EVA1028  *L-EVA1029*  L-EVA1089  L-EVA1090  *L-EVA1091* | 1.00 ± 0.04  0.94 ± 0.05  1.01 ± 0.05  1.00 ± 0.05  0.96 ± 0.06 |

**Table S4.** Moisture contents (and incorporated uncertainties) of the luminescence dating samples.

| **Sample** | **H_2_O (%)** |
| --- | --- |
| L-EVA1028  *L-EVA1029*  L-EVA1089  L-EVA1090  *L-EVA1091* | 10 ± 5  14 ± 5  17 ± 5  17 ± 5  17 ± 5 |

**Environmental magnetism**

**Table S5.** Mass specific and frequency dependent magnetic susceptibility of samples from subsections SS1 (NW profile) and SS2 (SE profile). Susceptibilities were measured in a field of 300 Am^-1^ at frequencies of 300 and 3000 Hz, respectively.

| **Sample name** | **Sample depth from tephra [m]** | **χ [kg^-1^*m^3^]** | **χ frequency dependent [%]** |
| --- | --- | --- | --- |
| **SS1 (NW profile)** | | | |
| SS1_0.00 | 3.60 | 3.54E-07 | 2.90 |
| SS1_0.05 | 3.55 | 3.50E-07 | 2.70 |
| SS1_0.10 | 3.50 | 3.45E-07 | 3.05 |
| SS1_0.15 | 3.45 | 3.42E-07 | 2.03 |
| SS1_0.20 | 3.40 | 3.49E-07 | 2.74 |
| SS1_0.25 | 3.35 | 3.46E-07 | 2.28 |
| SS1_0.30 | 3.30 | 3.38E-07 | 2.84 |
| SS1_0.35 | 3.25 | 3.37E-07 | 3.17 |
| SS1_0.40 | 3.20 | 3.39E-07 | 3.00 |
| SS1_0.45 | 3.15 | 3.35E-07 | 3.57 |
| SS1_0.50 | 3.10 | 3.37E-07 | 3.16 |
| SS1_0.55 | 3.05 | 3.31E-07 | 2.59 |
| SS1_0.60 | 3.00 | 3.31E-07 | 2.66 |
| SS1_0.65 | 2.95 | 3.38E-07 | 3.20 |
| SS1_0.70 | 2.90 | 3.31E-07 | 2.60 |
| SS1_0.75 | 2.85 | 3.39E-07 | 2.94 |
| SS1_0.80 | 2.80 | 3.35E-07 | 2.95 |
| SS1_0.85 | 2.75 | 3.38E-07 | 2.84 |
| SS1_0.90 | 2.70 | 3.39E-07 | 2.76 |
| SS1_0.95 | 2.65 | 3.49E-07 | 4.04 |
| SS1_0.100 | 2.60 | 3.51E-07 | 4.47 |
| SS1_0.105 | 2.55 | 3.51E-07 | 4.19 |
| SS1_0.110 | 2.50 | 3.48E-07 | 3.82 |
| SS1_0.115 | 2.45 | 3.38E-07 | 2.76 |
| SS1_0.120 | 2.40 | 3.42E-07 | 2.71 |
| SS1_0.125 | 2.35 | 3.40E-07 | 3.35 |
| SS1_0.130 | 2.30 | 3.51E-07 | 3.69 |
| SS1_0.135 | 2.25 | 3.46E-07 | 3.72 |
| SS1_0.140 | 2.20 | 3.65E-07 | 3.70 |
| SS1_0.145 | 2.15 | 3.64E-07 | 3.20 |
| SS1_0.150 | 2.10 | 3.76E-07 | 3.48 |
| SS1_0.155 | 2.05 | 3.80E-07 | 3.90 |
| SS1_0.160 | 2.00 | 3.87E-07 | 3.98 |
| SS1_0.165 | 1.95 | 3.96E-07 | 4.17 |
| SS1_0.170 | 1.90 | 3.91E-07 | 4.21 |
| SS1_0.175 | 1.85 | 3.88E-07 | 4.17 |
| SS1_0.180 | 1.80 | 3.86E-07 | 4.65 |
| SS1_0.185 | 1.75 | 3.88E-07 | 4.02 |
| SS1_0.190 | 1.70 | 3.90E-07 | 4.50 |
| SS1_0.195 | 1.65 | 3.88E-07 | 4.07 |
| SS1_0.200 | 1.60 | 3.97E-07 | 4.74 |
| SS1_0.205 | 1.55 | 4.02E-07 | 4.65 |
| SS1_0.210 | 1.50 | 4.04E-07 | 4.53 |
| SS1_0.215 | 1.45 | 4.00E-07 | 4.58 |
| SS1_0.220 | 1.40 | 3.99E-07 | 4.48 |
| SS1_0.225 | 1.35 | 3.98E-07 | 4.43 |
| SS1_0.230 | 1.30 | 3.99E-07 | 4.75 |
| SS1_0.235 | 1.25 | 4.16E-07 | 4.45 |
| SS1_0.240 | 1.20 | 4.18E-07 | 4.45 |
| SS1_0.245 | 1.15 | 4.15E-07 | 4.77 |
| SS1_0.250 | 1.10 | 4.05E-07 | 4.64 |
| SS1_0.255 | 1.05 | 4.21E-07 | 4.75 |
| SS1_0.260 | 1.00 | 4.33E-07 | 5.17 |
| SS1_0.265 | 0.95 | 4.33E-07 | 4.88 |
| SS1_0.270 | 0.90 | 4.94E-07 | 5.32 |
| SS1_0.275 | 0.85 | 4.42E-07 | 5.46 |
| SS1_0.280 | 0.80 | 4.61E-07 | 5.58 |
| SS1_0.285 | 0.75 | 5.20E-07 | 5.79 |
| SS1_0.290 | 0.70 | 5.17E-07 | 5.73 |
| SS1_0.295 | 0.65 | 5.01E-07 | 5.49 |
| SS1_0.300 | 0.60 | 5.31E-07 | 5.87 |
| SS1_0.305 | 0.55 | 5.35E-07 | 5.82 |
| SS1_0.310 | 0.50 | 5.65E-07 | 5.95 |
| SS1_0.315 | 0.45 | 5.31E-07 | 5.54 |
| SS1_0.320 | 0.40 | 5.06E-07 | 5.39 |
| SS1_0.325 | 0.35 | 4.77E-07 | 4.52 |
| SS1_0.330 | 0.30 | 5.45E-07 | 5.78 |
| SS1_0.335 | 0.25 | 5.41E-07 | 5.52 |
| SS1_0.340 | 0.20 | 1.48E-06 | 6.04 |
| SS1_0.345 | 0.15 | 9.28E-07 | 6.26 |
| SS1_0.350 | 0.10 | 1.54E-06 | 6.65 |
| SS1_0.355 | 0.05 | 6.48E-07 | 6.10 |
| SS1_0.360 | 0.00 | 6.70E-07 | 5.83 |
| *Stratigraphic position of tephra* | | | |
| **SS2 (SE profile)** | | | |
| *Stratigraphic position of tephra* | | | |
| SS2_0.00 | 0.00 | 4.23E-07 | 5.17 |
| SS2_0.05 | 0.05 | 4.66E-07 | 5.60 |
| SS2_0.10 | 0.10 | 4.17E-07 | 5.29 |
| SS2_0.15 | 0.15 | 4.03E-07 | 4.78 |
| SS2_0.20 | 0.20 | 4.50E-07 | 5.22 |
| SS2_0.25 | 0.25 | 4.23E-07 | 5.27 |
| SS2_0.30 | 0.30 | 4.13E-07 | 4.98 |
| SS2_0.35 | 0.35 | 4.11E-07 | 5.42 |
| SS2_0.40 | 0.40 | 4.14E-07 | 5.23 |
| SS2_0.45 | 0.45 | 4.17E-07 | 5.65 |
| SS2_0.50 | 0.50 | 4.14E-07 | 4.90 |
| SS2_0.55 | 0.55 | 4.12E-07 | 4.67 |
| SS2_0.60 | 0.60 | 4.12E-07 | 5.13 |
| SS2_0.65 | 0.65 | 4.11E-07 | 4.98 |
| SS2_0.70 | 0.70 | 4.12E-07 | 5.49 |
| SS2_0.75 | 0.75 | 4.16E-07 | 5.18 |
| SS2_0.80 | 0.80 | 4.12E-07 | 5.22 |
| SS2_0.85 | 0.85 | 4.22E-07 | 5.03 |
| SS2_0.90 | 0.90 | 4.07E-07 | 5.20 |
| SS2_0.95 | 0.95 | 3.98E-07 | 4.71 |
| SS2_0.100 | 1.00 | 4.17E-07 | 7.15 |
| SS2_0.105 | 1.05 | 4.12E-07 | 8.79 |
| SS2_0.110 | 1.10 | 4.08E-07 | 5.72 |
| SS2_0.115 | 1.15 | 4.07E-07 | 5.51 |
| SS2_0.120 | 1.20 | 4.07E-07 | 5.95 |
| SS2_0.125 | 1.25 | 4.34E-07 | 5.05 |
| SS2_0.130 | 1.30 | 4.37E-07 | 5.65 |
| SS2_0.135 | 1.35 | 4.24E-07 | 5.16 |
| SS2_0.140 | 1.40 | 4.04E-07 | 4.49 |
| SS2_0.145 | 1.45 | 4.02E-07 | 5.13 |
| SS2_0.150 | 1.50 | 3.93E-07 | 5.18 |
| SS2_0.155 | 1.55 | 3.81E-07 | 5.11 |
| SS2_0.160 | 1.60 | 3.75E-07 | 5.49 |
| SS2_0.165 | 1.65 | 3.64E-07 | 4.86 |
| SS2_0.170 | 1.70 | 3.63E-07 | 4.72 |
| SS2_0.175 | 1.75 | 3.75E-07 | 5.32 |
| SS2_0.180 | 1.80 | 3.78E-07 | 5.40 |
| SS2_0.185 | 1.85 | 3.86E-07 | 5.48 |
| SS2_0.190 | 1.90 | 3.66E-07 | 4.71 |
| SS2_0.195 | 1.95 | 3.80E-07 | 5.40 |
| SS2_0.200 | 2.00 | 3.86E-07 | 5.60 |
| SS2_0.205 | 2.05 | 3.87E-07 | 4.88 |
| SS2_0.210 | 2.10 | 3.83E-07 | 4.45 |
| SS2_0.215 | 2.15 | 3.44E-07 | 3.96 |
| SS2_0.220 | 2.20 | 3.24E-07 | 4.75 |
| SS2_0.225 | 2.25 | 3.47E-07 | 4.30 |
| SS2_0.230 | 2.30 | 5.00E-07 | 4.21 |
| SS2_0.235 | 2.35 | 4.01E-07 | 4.20 |
| SS2_0.240 | 2.40 | 3.75E-07 | 5.10 |
| SS2_0.245 | 2.45 | 3.73E-07 | 4.37 |
| SS2_0.250 | 2.50 | 4.01E-07 | 4.46 |
| SS2_0.255 | 2.55 | 4.48E-07 | 4.48 |
| SS2_0.260 | 2.60 | 3.26E-07 | 5.67 |
| SS2_0.265 | 2.65 | 3.82E-07 | 4.70 |
| SS2_0.270 | 2.70 | 3.36E-07 | 4.64 |
